# Statistics and Conclusions

**Published:** 1921-09-10

**Authors:** 


### THE LONDON COUNTY COUNCIL HEALTH REPORT.

### STATISTICS AND CONCLUSIONS.

This publication for 1920 is presented to the reader under seven headings, which are respectively, vital statistics and administration, school work, general public health, drainage, housing, financial, and venereal disease. A vast amount of information is naturally to be expected; and, on the whole, it is of a fairly satisfactory nature, that adjective being used, of course, in a relative sense, relative to "the measure of the possible." The marriage-rate is unaltered, but the birth-rate has risen, and the death-rate fallen, especially the infant death-rate. Agreeably with experience elsewhere, and in opposition to the doctrine of contagionists and bacteriologists, the main anti-tuberculosis factor is designated to be raising of individual resistance, not precautions against real or supposed infection. Smallpox is shown to be a constant, if not very great risk; but we think that the author is not correct in claiming that the Philippine Islands have been almost entirely rid of that disease. The usual zymotics have all increased, indeed the autumnal incidence of scarlet fever in London in 1920 was greater than in any year since 1892. There was no plague and no cholera, but fifteen cases of anthrax, four of them due to shaving brushes. The supposed "new disease" encephalitis lethargica, of newspaper celebrity, was notified to the extent of 149 cases. Dr. J. G. Forbes confirmed the diagnosis in 130 of these, and the mortality was close upon 37 per cent. Typhoid fever is the object of a careful statistical study, which lends "no support to a hypothesis that the fact that between 1914 and 1918 some four millions of men were inoculated against typhoid fever, has been a prime factor in reducing mortality." Nor (another blow to bacteriological confidence) is Dr. Hamer much impressed by the *rôle* of the "carrier" in the epidemiology of either this disease or dysentery.

### The Working-class Child.

In the field of educational medicine it is pleasant to note that the proportion of children found at routine inspections to require treatment for defects shows a marked decline. Various attempts have been made to do something to cope with the disabilities of the working-class child. At Woolwich a "tonsils and adenoids" centre has been opened at which children can be kept for two days after the operation. In Camberwell and Kensington the Borough Councils have lent hot-bathing accommodation. What is needed is extra measures against under-nutrition, for 9 per cent. of the boys at the age of eight are marked as under-nourished. This phenomenon, which has increased during the last two years, is attributed to the combined effects of high prices and of unemployment. There seem to be but three open-air day schools for children with consumption or scrofula. The estimate of the prevalence of definite pulmonary tubercle among school children appears decidedly optimistic, although the tuberculous officers are being encouraged to enter the schools.

### Housing Estimated.

The housing scheme, as finally approved, desiderates the provision within five years of 29,000 new dwellings, accommodating approximately 145,000 persons; 10,000 of the houses (serving 50,000 persons) to be completed within two years. The sites are at Hammersmith, Croydon, Tottenham, Roehampton, Lewisham, Dagenham, Bromley, and Barnes. At one of these places the tender accepted provided for the payment to the contractor of a fee of nearly half-a-million sterling. Another successful contractor, in connection with the Lewisham site, quoted a fee of £125,000. This firm's estimate (the lowest received) for four typical cottages, amounted to £4,148. The figure is about that prevalent in the North of England when private firms are engaged in the work, but a good deal higher than the cost when building guilds (bodies which now enjoy the support of the Wholesale Co-operative Society) are engaged. We see no mention of them here.

### Crowded V.D. Clinics.

The venereal diseases scheme is in essence the financial support of the "V.D. clinics" at the various more important hospitals in London and the suburbs. These centres have established other anti-venereal agencies and agents, such as hostels and rescue-workers. Much good is being done, and more still will be accomplished when the great hindrance of patients ceasing too soon their attendance, has been made less. To the onlooker it has been a joyful surprise, however, that infected people do not shy at coming to these places, publicly situated as some of them are. Indeed, many clinics are overcrowded. The filling of waiting-rooms for any length of time, especially waiting-rooms for women, has been found undesirable as leading to undecorous conversation. *Per contra*, however, we believe it is the case that in clinics serving small areas the infective prostitutes, or semi-prostitutes, or seducers, thus become generally known and avoidable.

### The M.O.H. and Authorship.

There is one feature of the report that strikes one in conclusion, and that is its length. It has 135 + xxxix very large pages; each page has about sixty lines, each line has about thirteen words. To save the reader the trouble of doing the sum, we might say that the publication is far longer than the ordinary novel. Why should Dr. Hamer, whose text is an enormous proportion of the whole, have to write a biggish book yearly? Must it not interfere with his work? Also, in view of the multifarious reference, which has perhaps already appeared, although far from sufficiently so, one's feeling as to the author is that still the wonder grows, how one small head can carry all he knows. Would it not be better, for the future, for the Medical Officer of Health to confine himself to general editorship, except as regards the general statistical survey, as to which (alone) he is a distinguished expert? With subordinates of the calibre, say, of Dr. Shrubsall, there would be no loss of quality. On the other hand, there should be an economy of space (which is especially valuable just now, with printing charges and money tightness what they are), because tuberculosis, for instance, could then be reported on, and done with, under one heading instead of appearing under more than one. The report would read better, too, in spite of the just possible loss of the tropes and quotations with which Dr. Hamer enlivens it; for order and arrangement please the attention like naught else. And, above all, an annual report in system form would encourage responsibility and keenness and the team spirit in officers with important clinical and administrative duties, those in whose hands the treatment and prevention of disease actually lie—and who, if the great mass of the people are to have proper medical succour, should be encouraged to the highest attainable clinical and investigatory standard.

